# Serum anti-PLA2R negativity does not exclude glomerular PLA2R expression in primary membranous nephropathy

**DOI:** 10.17305/bb.2026.14167

**Published:** 2026-06-08

**Authors:** Şenay Yıldırim, Ayça İnci, Ayşe Karaduru Avcı, Lütfullah Zahit Koç, Döndü Nergiz, Üstün Yılmaz

**Affiliations:** 1 Department of Pathology, Antalya Training and Research Hospital, Antalya, Türkiye; 2 Department of Internal Medicine, Division of Nephrology, Akdeniz University Faculty of Medicine, Antalya, Türkiye; 3 Department of Internal Medicine, Antalya City Hospital, Antalya, Türkiye; 4 Department of Internal Medicine, Serik State Hospital, Antalya, Türkiye; 5 Department of Pathology, Antalya Training and Research Hospital, Antalya, Türkiye; 6 Department of Internal Medicine, Division of Nephrology, The University of Health Sciences, Antalya Training and Research Hospital, Antalya, Türkiye

**Keywords:** Primary membranous nephropathy, anti-PLA2R antibodies, kidney-as-a-sink hypothesis, renal biopsy, glomerular PLA2R expression

## Abstract

Primary membranous nephropathy (pMN) is a principal cause of nephrotic syndrome in adults. The identification of the M-type phospholipase A2 receptor (PLA2R) antigen has significantly advanced non-invasive management; however, the precise clinical relationship between circulating antibody titers and intrarenal antigen deposition continues to be debated. This single-center retrospective study sought to analyze the correlation between clinicopathological parameters, serum anti-PLA2R levels, and glomerular PLA2R tissue expression in pMN. A specific focus was placed on evaluating the diagnostic utility of tissue staining in seronegative patients. A cohort of 49 adult pMN patients, diagnosed via renal biopsy between 2018 and 2025, was evaluated. Serum anti-PLA2R antibodies were quantified using ELISA, while glomerular PLA2R expression and staining intensity (graded 0 to +3) were assessed via immunohistochemistry (IHC) on paraffin-embedded sections. The results demonstrated a notable discordance: the overall serum antibody positivity rate was 49.0%, yet tissue PLA2R expression was detected in 100% of the cohort, encompassing all seronegative cases. A statistically significant difference was observed in the distribution of tissue PLA2R staining intensity based on serum PLA2R status (*P ═* 0.002). Conversely, no statistically significant correlation was found between circulating antibody titers and baseline renal function or proteinuria markers (*P >* 0.05). In conclusion, these findings indicate that negative serology does not preclude tissue PLA2R positivity, potentially attributable to mechanisms such as the “kidney-as-a-sink” phenomenon or persistent immunological footprints. This investigation underscores that serum and tissue PLA2R serve as complementary, rather than mutually exclusive, markers. Consequently, renal biopsy with supplementary IHC staining remains a crucial and clinically valuable diagnostic tool, particularly in seronegative cases.

## Introduction

Membranous nephropathy (MN) is a prevalent cause of nephrotic syndrome in adults, characterized by podocyte injury and subepithelial immune complex deposition within the glomerular basement membrane (GBM) [1, 2]. Morphological hallmarks include diffuse GBM thickening on light microscopy and “spike” formations detected by silver staining [3]. Traditionally, MN is categorized into primary membranous nephropathy (pMN), accounting for approximately 80% of cases with no identifiable etiology, and secondary MN, associated with infections, malignancies, systemic autoimmune diseases, or drug exposure [1, 4].

The identification of the M-type phospholipase A2 receptor (PLA2R) as a major target antigen on the podocyte surface by Beck et al. in 2009 revolutionized MN classification [4]. This discovery facilitated the use of a serological biomarker for diagnosing, prognostically assessing, and therapeutically monitoring pMN [5, 6]. Serum anti-PLA2R antibodies can be detected using immunofluorescence assay (IFA), enzyme-linked immunosorbent assay (ELISA), and Western blot techniques [7]. For patients seronegative for anti-PLA2R antibodies, evaluating PLA2R expression at the tissue level via immunohistochemistry (IHC) and immunofluorescence (IF) addresses this diagnostic gap [8]. While a positive serum anti-PLA2R result is a robust diagnostic tool for pMN, its exclusive use remains contentious due to concerns regarding potential misclassification of non-pMN cases or loss of prognostic information if biopsy is deferred. Consequently, the necessity of renal biopsy continues to be debated [7].

Over the past decade, numerous novel antigens have been identified in MN biopsy specimens, particularly in adult cohorts. These include thrombospondin type-1 domain-containing 7A (THSD7A), exostosin 1 and 2 (EXT1/2), neural epidermal growth factor-like 1 (NELL1), protocadherin 7, neural cell adhesion molecule 1 (NCAM1), semaphorin 3B (SEMA3B), transforming growth factor-beta receptor 3 (TGFBR3), high-temperature requirement A serine peptidase 1 (HTRA1), neuron-derived neurotrophic factor, and protocadherin Fat 1 (FAT1), among others [8, 9]. Although PLA2R remains the most common target antigen, MN cases associated with these newly identified antigens exhibit distinct clinical and pathological features, necessitate varied therapeutic strategies, and have diverse prognostic outcomes. Currently, serological assays are primarily available for PLA2R and THSD7A [8, 10].

The clinical trajectory of pMN is highly variable; approximately one-third of patients experience spontaneous remission, while another one-third progress to end-stage renal disease without specific treatment [11]. Treatment decisions have historically relied on proteinuria levels and estimated glomerular filtration rate (eGFR). However, there is an ongoing need for reliable biomarkers to mitigate the adverse effects of immunosuppressive therapy and enable personalized treatment strategies. While serum anti-PLA2R levels are reported to reflect immunological activity, seronegativity can occur, especially in early-stage disease or when antibodies are sequestered within renal tissue [10, 12].

The primary objective of this study was to analyze the relationship between clinicopathological parameters, serum anti-PLA2R levels, and tissue PLA2R expression in renal biopsies from pMN patients. Specifically, we aimed to investigate the diagnostic utility of tissue staining in seronegative cases and to evaluate the complementary roles of these two markers in clinical practice.

## Materials and methods

### Study population

**Figure 1.**
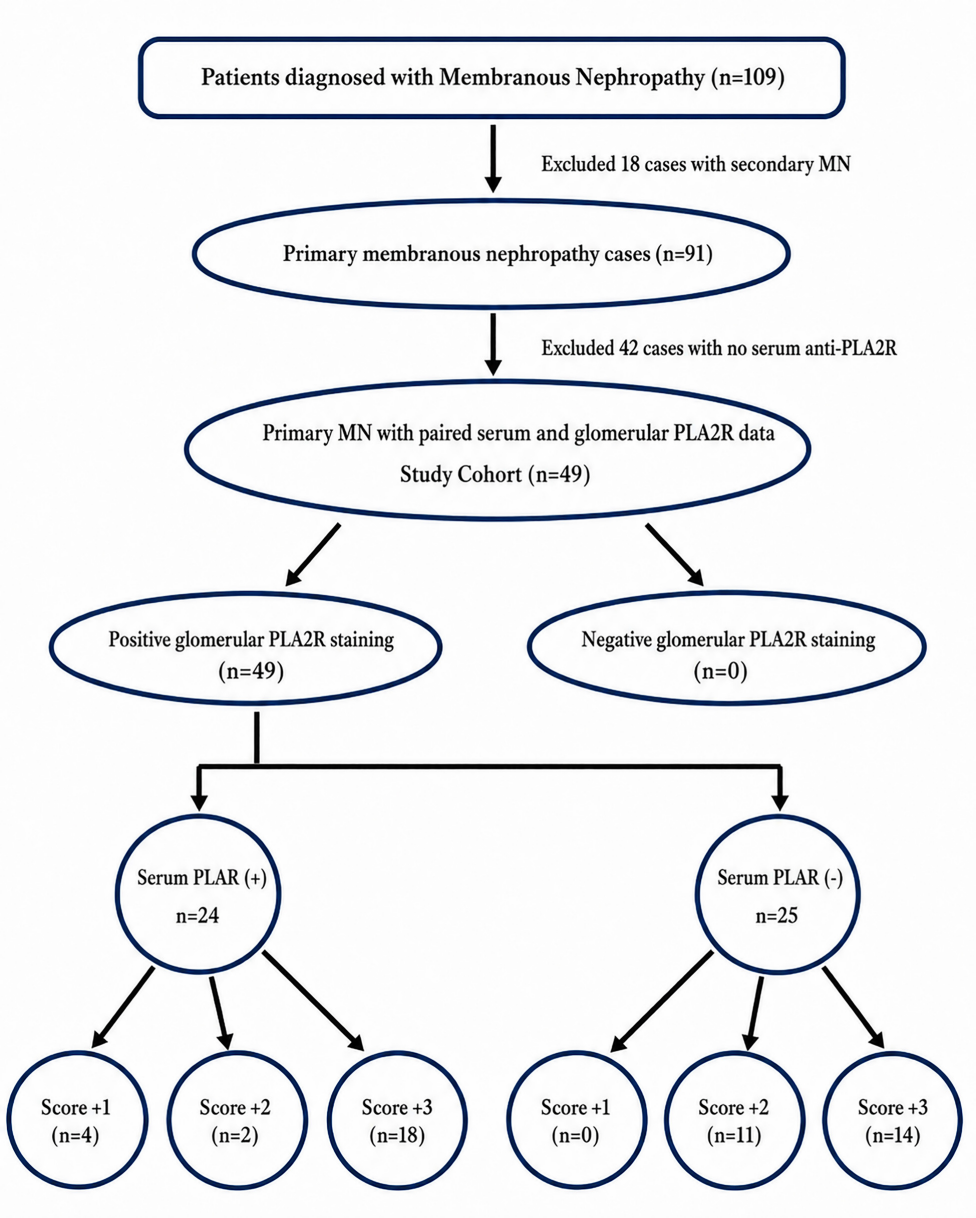
Eligibility and exclusion criteria for the study population.

This retrospective study analyzed consecutively screened adult patients diagnosed with MN via renal biopsy between January 2018 and December 2025. Among patients classified as pMN after excluding secondary causes, only those with available serum anti-PLA2R measurements obtained within 15 days prior to renal biopsy and tissue PLA2R staining data were included. The primary reason for excluding otherwise eligible pMN cases was the unavailability of serum anti-PLA2R testing, particularly in earlier years of the study period when the assay was not routinely accessible or reimbursed (Figure 1).

### Clinical and laboratory assessment

Demographic data (age, sex) and comorbidities (hypertension (HT), diabetes mellitus (DM)) were extracted from medical records. Laboratory parameters evaluated at diagnosis included blood urea nitrogen (BUN), serum creatinine (Scr), and serum albumin. Urinary protein excretion was concurrently assessed using both spot urine samples—specifically spot urine protein-to-creatinine ratio (UPCR) and spot albumin-to-creatinine ratio (UACR)—and 24-hour urine collections to measure 24-hour total urine protein and 24-hour UACR. Laboratory measurements were obtained either at the time of renal biopsy or within a maximum of 15 days prior to the procedure, before the initiation of any immunosuppressive therapy. The eGFR was calculated using the Chronic Kidney Disease Epidemiology Collaboration (CKD-EPI) equation.

**Table 1:** Comparison of demographic and clinical characteristics of patients according to serum PLA2R status

	**Serum PLA2R negative**		**Serum PLA2R positive**		
**Parameter**	***n***	**Mean±SD (min-max)**	***n***	**Mean±SD (Min-Max)**	***P* value**
		**Median (Q_1_-Q_3_)**		**Median (Q_1_-Q_3_)**	
Age at diagnosis (years)	25	54±13 (30--77)	24	52±14 (28--76)	0,657^1^
Serum creatinine (mg/dl)	25	0,97 (0,8--1,14)	24	1,08 (0,88--1,26)	0,23^2^
Serum albumin (mg/dl)	25	2,61±0,79 (1,3--4)	24	2,6±0,6 (1,5--3,8)	0,953^1^
eGFR (ml/min/1.73m^2^)	25	84 (61--93)	24	70 (59,5--94)	0,509^2^
24h UACR (mg/24h)	16	5493,62±2617,13 (950--11959)	9	5235,78±4489,84 (45--14517)	0,857^1^
24h UP (mg/24h)	16	8997,88±5187,4 (1551--21558)	10	9055,4±7143,16 (1598--22375)	0,981^1^
Spot albumin/Cr (mg/g)	16	5506,19±4506,26 (157--16649)	18	5652,44±3249,38 (1141--13987)	0,914^1^
Spot protein/Cr (mg/g)	17	9221,65±7197,62 (812--25117)	21	9451±5618,54 (1000--24123)	0,913^1^
Most recent eGFR (ml/min/1.73m^2^)	22	77,41±27,11 (23--121)	22	62,41±31,49 (8--109)	0,098^1^
Most recent albumin (mg/dl)	22	3,8 (3,5--4,2)	22	3,7 (3,2--4,1)	0,621^2^
Most recent proteinuria (mg/dl)	22	872,5 (272--2854)	22	1943 (888--4720)	0,159^2^
Follow-up time (months)	22	16 (7--33)	22	23,5 (11--56)	0,127^2^
Female		12 (48%)		6 (25%)	0,095^3^
Male		13 (52%)		18 (75%)	
HT (-)		9 (36%)		2 (8,3%)	0,02^3^
HT (+)		16 (64%)		22 (91,7%)	
DM (-)		22 (88%)		20 (83,3%)	0,702^4^
DM (+)		3 (12%)		4 (16,7%)	

^1^The independent two-sample *t*-test, ^2^Mann–Whitney *U* test, ^3^Pearson’s chi-square test, and ^4^Fisher’s exact test were used for statistical analyses. Continuous variables are presented as mean±SD (min–max) or median (Q1–Q3), whereas categorical variables are expressed as count (percentage). Abbreviations: Cr, creatinine; DM, diabetes mellitus; eGFR, estimated glomerular filtration rate; HT, hypertension; n, number of patients; PLA2R, phospholipase A2 receptor; Q1–Q3, first to third quartile; SD, standard deviation; UACR, urinary albumin-to-creatinine ratio; UP, urinary protein.

A comprehensive evaluation was performed to exclude secondary causes in patients diagnosed with MN. This included serological and laboratory tests (antinuclear antibodies [ANA], anti-double-stranded DNA antibodies [anti-dsDNA], complement levels, hepatitis and human immunodeficiency virus [HIV] panels, TSH, and Free T4), a review of medication histories, and malignancy screenings tailored to the patient’s age group and presenting symptoms. Within the scope of screening, all patients underwent chest X-ray and abdominal ultrasonography; additionally, mammography and PAP smear were performed for female patients, while PSA levels were monitored for male patients.

### Serological analysis

Serum anti-PLA2R antibody levels were measured using a commercial ELISA kit (Euroimmun AG, Germany) prior to renal biopsy. Results were categorized as negative (<20 RU/mL) or positive (≥20 RU/mL) [13].

### Histopathological, immunohistochemical, and IF examination

Renal biopsy specimens were evaluated using standard staining methods, including Hematoxylin & Eosin, Masson’s trichrome, Periodic Acid–Schiff (PAS), Jones methenamine silver, and Congo red stains. For IF analysis, a panel including IgG, IgA, IgM, kappa, lambda, C3, and C1q antisera was used. In cases where IF analysis was not feasible due to inadequate tissue sampling or fixation artifacts, IHC staining for IgG, IgA, IgM, and kappa and lambda light chains was performed on formalin-fixed paraffin-embedded (FFPE) tissue blocks. The demonstration of granular immune complexes and light chain deposition along the GBM provided robust diagnostic confirmation for MN.

Immunohistochemical staining for PLA2R (ready-to-use; BSB-2372-7; Bio SB, USA) was performed on paraffin-embedded sections using the Roche Ventana Benchmark Ultra system. PLA2R staining intensity was independently evaluated by two blinded pathologists. Full inter-observer agreement (100% consensus) was achieved in all cases, thus eliminating the need for the pre-planned re-staining protocol for discrepant results. Staining intensity was assessed based on the intensity and continuity of granular staining along the GBM. Scores were assigned as follows: 0 (no staining), +1 (weak, thin, discontinuous granular staining), +2 (moderate, continuous granular staining), and +3 (strong, thick, coarse granular staining) [14]. Specimens lacking glomeruli in IF sections were considered inadequate, whereas those without immunoglobulin or complement deposition were considered negative.

### Ethical statement

This study received approval from the Scientific Research Ethics Committee of Antalya Training and Research Hospital (Decision No: 21/16, Date of Approval: December 11, 2025).

### Statistical analysis

Descriptive statistics were presented as frequencies and percentages for categorical variables, and as mean ± standard deviation (minimum–maximum) or median (interquartile range [IQR], Q1–Q3) for continuous variables, depending on their distribution. The Shapiro-Wilk test was used to evaluate the assumption of normality for continuous variables. For the analysis of differences in numerical data between two groups, the Independent Samples *t*-test was employed for normally distributed data, and the Mann-Whitney *U* test for non-normally distributed data.

**Table 2:** Correlation between serum PLA2R levels and clinical parameters

	**Age at diagnosis (years)**	**Cr (mg/dl)**	**BUN (mg/dl)**	**Alb (mg/dl)**	**eGFR (ml/min/1.73m^2^)**	**24 h UACR (mg/24h)**	**24 h UP** **(mg/24h)**	**Spot Alb/Cr (mg/g)**	**Spot P/Cr (mg/g)**	**Most recent eGFR (ml/min/1.73m^2^)**	**Most recent albumin (mg/dl)**	**Most recent proteinuria (mg/dl)**
**Serum PLA2R level**												
**r**	0,022	--0,078	--0,082	--0,008	--0,073	--0,35	--0,309	0,067	0,171	--0,023	--0,157	0,28
***P***	0,92	0,717	0,702	0,971	0,733	0,356	0,385	0,791	0,457	0,921	0,485	0,208
***n***	24	24	24	24	24	9	10	18	21	22	22	22

Abbreviations: Alb, albumin; Alb/Cr, albumin-to-creatinine ratio; BUN, blood urea nitrogen; Cr, creatinine; eGFR, estimated glomerular filtration rate; n, number of patients; P/Cr, protein-to-creatinine ratio; PLA2R, phospholipase A2 receptor; r, correlation coefficient; UACR, urinary albumin-to-creatinine ratio; UP, urinary protein.

**Figure 2.**
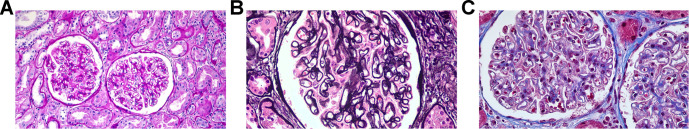
**Light microscopic features of primary membranous nephropathy.** (A) PAS staining shows diffuse thickening of the GBMs, a characteristic light microscopic finding in membranous nephropathy (original magnification, ×200). (B) JMS staining highlights characteristic spike formations along the GBM, corresponding to new basement membrane material extending between subepithelial deposits (original magnification, ×400). (C) Masson’s trichrome staining demonstrates fuchsinophilic granular deposits along the thickened GBM, consistent with subepithelial immune complex deposition (original magnification, ×400). Abbreviations: GBM, glomerular basement membrane; JMS, Jones methenamine silver; PAS, Periodic acid–Schiff.

In the analysis of relationships between categorical variables, Fisher’s Exact Test and the Fisher-Freeman-Halton Exact Test were utilized if more than 20% of the cells had an expected count of less than 5; otherwise, the Pearson Chi-Square Test was employed. For categorical relationships between serum anti-PLA2R status/titer groups and tissue PLA2R staining grade, the effect size was expressed using Cramer’s V.

The relationships between serum anti-PLA2R titer levels and clinical or laboratory variables were assessed using the non-parametric Spearman’s rank correlation coefficient. These correlations were limited exclusively to serum anti-PLA2R-positive patients with measurable titer values (n ≤ 24) and were further constrained for 24-hour urine parameters due to incomplete collection. Given the small number of observations for certain variables, these correlations were interpreted with caution and considered exploratory. Proportions regarding serum-tissue discordance were presented as n/N, percentages, and exact binomial (Clopper-Pearson) 95% confidence intervals.

All statistical analyses were performed using IBM SPSS Statistics version 30. A two-tailed *P*-value of < 0.05 was considered statistically significant.

## Results

### Baseline characteristics and clinicopathological comparisons

Table 1 presents the demographic and clinicopathological characteristics of 49 pMN patients, categorized by their serum PLA2R status. Of the 49 patients analyzed, 25 (51%) were in the serum PLA2R-negative group, and 24 (49%) were in the serum PLA2R-positive group. The median age at diagnosis was 55 years for serum PLA2R-negative patients and 51 years for serum PLA2R-positive patients, demonstrating no statistically significant difference between groups (*P* ═ 0.657). While gender distribution did not reach statistical significance (*P* ═ 0.095), a higher male predominance was observed in the serum PLA2R-positive group (75%) compared to the negative group (52%) (Table 1).

### Biochemical and renal function parameters

At admission, no significant differences were found between serum PLA2R-negative and positive groups regarding key renal function markers and biochemical parameters, including creatinine, albumin, and eGFR (*P* > 0.059). Similarly, proteinuria levels, assessed by 24-hour UACR, 24-hour total urinary protein, and spot urine protein-to-creatinine or albumin-to-creatinine ratios, did not differ significantly between the groups (*P* > 0.05) (Table 1).

### Comorbidities and follow-up outcomes

The prevalence of HT was 64.0% in the serum PLA2R-negative group and 91.7% in the serum PLA2R-positive group. Conversely, normotensive patients constituted 36.0% and 8.3% of these groups, respectively. No significant difference was observed in the prevalence of DM between the groups (16.7% in the positive group vs. 12% in the negative group; *P* ═ 0.702). At the conclusion of the follow-up period, there were no significant differences between the two groups in terms of final eGFR, final albumin levels, final proteinuria levels, or the total duration of follow-up (*P* > 0.05) (Table 1).

Spearman’s correlation analysis was employed to evaluate the relationships between serum PLA2R levels and various clinical and laboratory parameters. No statistically significant correlation was observed between serum PLA2R levels and any of the investigated variables (*P* > 0.05). Detailed correlation coefficients and *P*-values for all parameters, including baseline renal functions, proteinuria markers, and follow-up outcomes, are presented in Table 2.

### Histopathological features

Light microscopy of all renal biopsy specimens consistently revealed diffuse thickening of the GBM, most clearly visualized with PAS staining (Figure 2A). Characteristic spike formations, indicative of new basement membrane synthesis extending between subepithelial deposits, were demonstrated using Jones methenamine silver staining (Figure 2B). Masson’s trichrome staining highlighted typical fuchsinophilic granular deposits within the subepithelial region of thickened GBMs (Figure 2C).

### IF accumulations and relationship with serum PLA2R

A statistically significant difference was observed in the distribution of IF staining between serum PLA2R-negative and positive patients (*P* ═ 0.036). While the rates of inadequate IF staining were similar between the groups (32% in the negative group vs. 29.2% in the positive group), the rate of negative IF staining was significantly higher in the serum PLA2R-negative group (36% vs. 8.3%). Conversely, the rate of positive IF staining was significantly higher in the serum PLA2R-positive group compared to the negative group (62.5% vs. 32%) (Table 3).

**Table 3:** Comparison of IF accumulation types based on serum PLA2R status

	**Serum PLA2R status**		
	**Negative *n* (%)**	**Positive *n* (%)**	**Total *n* (%)**	***p***
**IF accumulation** **types**				
**Inadequate**	8a (32%)	7a (29,2%)	15 (30,6%)	0,036
**Negative**	9a (36%)	2b (8,3%)	11 (22,4%)	
**Positive**	8a (32%)	15b (62,5%)	23 (46,9%)	
**Total**	25 (100%)	24 (100%)	49 (100%)	

Pearson’s chi-square test was used. Different letters across a row indicate statistically significant differences between column proportions (*P* < 0.05). Abbreviations: IF, immunofluorescence; n, number of patients; PLA2R, phospholipase A2 receptor.

### Comparison of serum PLA2R levels and glomerular PLA2R expression

**Figure 3.**
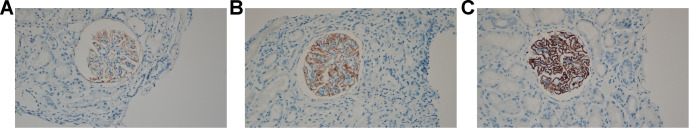
**IHC grading of glomerular PLA2R expression in primary membranous nephropathy.** (A) Grade +1 staining shows weak, thin, and focally discontinuous granular PLA2R positivity along the glomerular capillary walls (original magnification, ×200). (B) Grade +2 staining demonstrates moderate and more continuous granular PLA2R expression along the glomerular capillary walls (original magnification, ×200). (C) Grade +3 staining shows strong, diffuse, and coarse granular PLA2R positivity, consistent with high-intensity tissue expression (original magnification, ×200). Abbreviations: IHC, immunohistochemistry; PLA2R, phospholipase A2 receptor.

Immunohistochemical PLA2R staining revealed positive staining of varying intensities in all renal biopsies. Specifically, 4 cases were graded as +1 (Figure 3A), 13 cases as +2 (Figure 3B), and 32 cases as +3 (Figure 3C). Among the 45 patients exhibiting high tissue expression (grades +2 and +3), 55.6% (*n* ═ 25) were seronegative for anti-PLA2R antibodies. Conversely, all 4 patients with low expression (grade +1) exhibited serum anti-PLA2R antibody positivity.

A statistically significant difference was observed in the distribution of tissue PLA2R staining intensity based on serum PLA2R status (*P* ═ 0.002). Grade 1 staining was exclusively observed in the serum PLA2R-positive group (16.7% vs. 0%). In contrast, the prevalence of Grade 2 staining was significantly higher in the serum PLA2R-negative group compared to the positive cohort (44% vs. 8.3%). While Grade 3 staining was the most common finding in both groups, its higher frequency in the serum PLA2R-positive group (75% vs. 56%) did not reach statistical significance (*P* > 0.05) (Table 4).

**Table 4:** Distribution of tissue PLA2R staining intensity according to serum anti-PLA2R antibody status

	**Serum PLA2R status**		
	**Negative *n* (%)**	**Positive** ***n* (%)**	**Total *n* (%)**	***p***
**PLA2R staining** **intensity**				
**Grade 1**	0a (0%)	4b (16,7%)	4 (8,2%)	0,002
**Grade 2**	11a (44%)	2b (8,3%)	13 (26,5%)	
**Grade 3**	14a (56%)	18a (75%)	32 (65,3%)	
**Total**	25 (100%)	24 (100%)	49 (100%)	

Fisher’s exact test was employed. Distinct letters within a row signify statistically significant differences between column proportions (*P* < 0.05). Abbreviation: PLA2R, phospholipase A2 receptor.

Table 5 summarizes serum anti-PLA2R and tissue PLA2R proportions. Serum anti-PLA2R positivity was detected in 24 of 49 patients (49.0%; 95% CI: 34.4%–63.7%), with 25 patients being serum anti-PLA2R negative (51.0%; 95% CI: 36.3%–65.6%). Tissue PLA2R staining was positive in all patients (49/49, 100.0%; 95% CI: 92.7%–100.0%), and Grade 3 tissue PLA2R staining was observed in 32 of 49 patients (65.3%; 95% CI: 50.4%–78.3%).

**Table 5:** Prevalence and serum-tissue discordance of anti-PLA2R antibodies and PLA2R expression

**Measure**	***n*/N**	**%**	**95% CI**
**Basic prevalence proportions**			
Serum anti-PLA2R positivity	24/49	49.0	34.4--63.7
Serum anti-PLA2R negativity	25/49	51.0	36.3--65.6
Tissue PLA2R expression positivity	49/49	100.0	92.7--100.0
Grade 3 tissue PLA2R expression positivity	32/49	65.3	50.4--78.3
**Serum-tissue discordance proportions**			
Overall serum-tissue discordance: serum anti-PLA2R negative/tissue PLA2R positive**^a^**	25/49	51.0	36.3--65.6
Tissue PLA2R positivity among serum anti-PLA2R-negative patients	25/25	100.0	86.3--100.0
Serum anti-PLA2R negativity among patients with Grade 2–3 tissue PLA2R expression positivity	25/45	55.6	40.0--70.4
Serum anti-PLA2R negativity among patients with Grade 3 tissue PLA2R expression positivity	14/32	43.8	26.4--62.3

Data are presented as *n*/N, percentage, and exact binomial (Clopper-Pearson) 95% CI. **^a^**Because tissue PLA2R positivity was present in all patients (100%), the overall serum-negativity proportion is numerically identical to the overall serum-tissue discordance proportion. Abbreviations: anti-PLA2R, anti-phospholipase A2 receptor antibody; CI, confidence interval; n/N, number of patients with the finding/total number of patients; PLA2R, phospholipase A2 receptor.

Overall serum-tissue discordance, defined as serum anti-PLA2R negativity despite positive tissue PLA2R staining, was present in 25 of 49 patients, representing 51.0% of the total cohort (95% CI: 36.3%–65.6%). Given that tissue PLA2R positivity was observed in all patients, this proportion was numerically identical to the overall serum anti-PLA2R negativity rate. Among serum anti-PLA2R-negative patients, tissue PLA2R positivity was observed in all cases (25/25, 100.0%; 95% CI: 86.3%–100.0%). Furthermore, among patients with Grade 2–3 tissue PLA2R staining, 25 of 45 were serum anti-PLA2R negative (55.6%; 95% CI: 40.0%–70.4%). Among patients with Grade 3 tissue PLA2R staining, 14 of 32 were serum anti-PLA2R negative (43.8%; 95% CI: 26.4%–62.3%).

In an exploratory analysis, tissue PLA2R staining intensity was compared across serum anti-PLA2R level groups (Table 6). The distribution of tissue PLA2R staining grades significantly differed across the groups as determined by the Fisher-Freeman-Halton exact test (*P* ═ 0.004), with a moderate effect size (Cramer’s *V* ═ 0.37). Grade 1 staining was observed in 0 of 25 serum anti-PLA2R-negative patients, 3 of 12 patients with serum anti-PLA2R levels ≤139 RU/mL, and 1 of 12 patients with serum anti-PLA2R levels >139 RU/mL.

All serum PLA2R-negative patients exhibited either Grade 2 or Grade 3 tissue staining. Specifically, the rate of Grade 2 staining in this group was significantly higher compared to the ≤139 RU/mL serum-positive group (44% vs. 0%). Grade 3 staining remained the predominant finding across all cohorts, irrespective of serum levels, showing no statistically significant intergroup variation.

## Discussion

MN is pathologically characterized by podocyte injury and glomerular basement membrane damage, resulting from *in situ* immune complex formation and complement activation against podocyte antigens [15, 16]. While significant progress has been made in understanding its pathogenesis over the past two decades, the precise relationship between serological markers and tissue findings remains incompletely elucidated [16, 17]. This study investigated the clinical, biochemical, and pathological characteristics of pMN patients categorized by their serum PLA2R status. Our findings revealed substantial phenotypic similarity between serum PLA2R-positive and negative patients concerning fundamental renal function parameters and proteinuria levels. These results suggest that systemic antibody titers may not accurately reflect current clinical disease severity. Notably, we observed 100% tissue PLA2R positivity across the entire cohort, including all seronegative cases. This finding underscores the diagnostic challenges posed by isolated serological assessments and emphasizes the critical role of renal biopsy in routine clinical practice for comprehensive MN diagnosis.

### Discordance between serum and glomerular PLA2R

Our study revealed a striking discrepancy between circulating anti-PLA2R antibodies and glomerular PLA2R antigen expression. Despite 100% tissue PLA2R expression across the cohort, serum anti-PLA2R positivity was only 49.0%. This aligns with a recent study reporting serum PLA2R positivity in 42% and glomerular PLA2R deposition in 86% of pMN patients, with 79.3% of tissue-positive cases being seronegative [18].

**Table 6:** Comparison of tissue PLA2R staining grades according to serum PLA2R levels

**Tissue PLA2R staining grade**	**Serum anti-PLA2R negative n (%)**	**≤139 n (%)**	**>139 n (%)**	**Total n (%)**	***P***	**Cramer’s V**
**Grade 1**	0 (0.0)	3 (25.0)	1 (8.3)	4 (8.2)	0.004	0.37
**Grade 2**	11 (44.0)	0 (0.0)	2 (16.7)	13 (26.5)		
**Grade 3**	14 (56.0)	9 (75.0)	9 (75.0)	32 (65.3)		
**Total**	25 (100.0)	12 (100.0)	12 (100.0)	49 (100.0)		

Data are presented as n (%). The *P* value was determined using the Fisher-Freeman-Halton exact test. Cramer’s V served as the effect size for the association between serum anti-PLA2R level groups and tissue PLA2R staining grade. Due to the small and imbalanced subgroup sizes, particularly for Grade 1 staining, this analysis is considered exploratory. Abbreviations: anti-PLA2R, anti-phospholipase A2 receptor antibody; n, number of patients; PLA2R, phospholipase A2 receptor.

Several mechanisms may account for the observed discordance between circulating anti-PLA2R levels and glomerular PLA2R deposition. First, serum anti-PLA2R levels reflect current immunological activity, whereas glomerular PLA2R deposition represents a persistent “immunological footprint” of past or ongoing immune complex formation [10, 18, 19]. Therefore, tissue staining may remain positive even after circulating antibodies have cleared or declined below detectable levels. Second, the “kidney-as-a-sink” hypothesis posits that antibodies produced during the initial stages of the disease preferentially bind to intrarenal targets, leading to their depletion from systemic circulation. Consequently, detectable serum levels may only emerge once renal binding sites are saturated, allowing excess antibodies to spill into the bloodstream [12].

Conversely, the approach proposed by Bobart et al. [20], which suggests omitting renal biopsy in patients with positive serum anti-PLA2R, risks overlooking concurrent or alternative renal pathologies. For example, in diabetic patients with nephrotic syndrome, while anti-PLA2R antibodies strongly suggest pMN, their presence does not exclude coexisting diabetic nephropathy or other non-diabetic kidney diseases, such as focal segmental glomerulosclerosis, acute tubular necrosis, or IgA nephropathy [21]. While the high specificity of serological assays offers substantial diagnostic utility, renal biopsy remains invaluable for establishing a diagnosis in patients with negative anti-PLA2R results [22].

In our study, HT was significantly more frequent in the serum anti-PLA2R-positive group (22/24, 91.7%) than in the serum anti-PLA2R-negative group (16/25, 64.0%; *P* ═ 0.020). For these individuals, renal biopsy also provides substantial clinical utility in detecting other underlying renal pathologies. However, this unadjusted, descriptive comparison of HT did not account for potential confounders such as age or chronic kidney disease (CKD) stage. Therefore, these findings should be interpreted descriptively rather than as evidence of an independent association.

No statistically significant relationships were detected between serum PLA2R levels and clinical or laboratory variables (*P* > 0.05). Given that several of these correlations were based on a small number of observations (*n* ═ 9–24), the absence of significant associations should be interpreted as exploratory and may reflect limited statistical power rather than a true absence of association.

Furthermore, considering the presence of negative results in routine IF staining (36% in the seronegative group), performing additional immunohistochemical staining (IgG, IgA, IgM, Kappa, Lambda, PLA2R) on biopsy samples is a valuable step in the diagnostic process.

### Association between anti-PLA2R titer and glomerular PLA2R staining intensity

The most striking finding of our study is that all serum-negative patients exhibited either grade 2 or grade 3 tissue staining, with grade 3 staining remaining the dominant pattern (56%) even in the completely seronegative subgroup. This high-intensity tissue expression in the absence of circulating antibodies suggests that peripheral blood titers may be misleading and may not fully reflect the true extent of immunological accumulation within the glomeruli.

Li et al. reported that patients with tissue PLA2R positivity but negative serum antibodies exhibited milder clinical features and more favorable outcomes [23]. However, our Spearman’s correlation analysis revealed no statistically significant correlation between serum anti-PLA2R titers and baseline renal function, proteinuria markers, or follow-up outcomes (*P* > 0.05). This lack of correlation suggests that standard laboratory biomarkers alone may be insufficient to predict or guide clinicians regarding the intrarenal antibody burden.

Upon classifying patients into three distinct groups based on their serum PLA2R titers (negative, ≤139 RU/mL, and >139 RU/mL), a statistically significant variation was identified in the distribution of glomerular PLA2R staining intensity (*P* ═ 0.004). Similarly, Gunda et al. observed a trend toward increasing antibody titers with greater staining intensity, although this did not reach statistical significance (*P* ═ 0.616) [18]. Sun et al. demonstrated that tissue antigen density was prognostically relevant in patients with lower antibody levels (<150 RU/mL), whereas serum antibody burden became the dominant prognostic factor at higher levels (≥150 RU/mL) [24].

### Limitations and future perspectives

Our study included only patients with matched serum anti-PLA2R measurements and tissue PLA2R staining data. This requirement may have introduced selection bias, particularly due to the lack of routine availability or consistent access to anti-PLA2R testing in earlier years of the study. Consequently, patients who underwent serological testing might represent a subgroup more intensively studied due to higher clinical suspicion, more severe disease, or better access to healthcare. Furthermore, as the study was conducted in a tertiary referral center, referral bias may have contributed to an overrepresentation of complex or atypical cases. Socioeconomic disparities affecting access to specialized serological testing may have further influenced cohort composition. Collectively, these factors may limit the generalizability of our findings to the broader pMN population and should be considered when interpreting the observed serum-tissue PLA2R discordance.

Although extensive clinical and laboratory evaluations, including age-appropriate malignancy screening, were performed to rule out secondary causes of MN, a fully standardized malignancy screening protocol could not be uniformly implemented across all patients due to the retrospective, single-center design. Additionally, there was no predefined minimum longitudinal follow-up duration specifically aimed at ruling out subsequently emerging occult malignancy-associated MN. Therefore, the possibility that a small subset of patients classified as pMN might have represented an early or subclinical phase of malignancy-associated disease cannot be entirely excluded.

Furthermore, given that anti-PLA2R testing is not reimbursed and remains inaccessible in many public facilities, our cohort may be skewed toward patients of higher socioeconomic standing. Although the cohort demonstrated relatively balanced baseline clinical and laboratory characteristics, the retrospective single-center design and the exclusion of patients without available serum anti-PLA2R testing should be considered when interpreting the representativeness of the study population. Due to the relatively small and unbalanced sample sizes within certain subgroups, the correlations between serum anti-PLA2R titers, tissue staining intensity, and clinical variables should be interpreted cautiously and considered exploratory.

Electron microscopy was not available at our institution during the study period and, therefore, was not routinely performed. Consequently, ultrastructural assessment of electron-dense deposits, pathological staging of MN, and detection of subtle concurrent glomerular abnormalities could not be undertaken. In the absence of electron microscopy, diagnosis was based on light microscopy, IF, and immunohistochemical findings. This limitation should be considered when interpreting the study results.

The primary reason for the high rate of insufficient IF findings observed in our single-center experience was attributed to an inadequate number of glomeruli in the fresh-frozen tissue samples allocated for IF analysis (e.g., samples consisting solely of medulla). Additionally, it is believed that the storage conditions of the fresh-frozen tissue, the transport process, or technical complications during the sectioning stage may have adversely affected signal intensity. However, immunohistochemical PLA2R antibody staining performed on sections prepared from paraffin blocks containing glomeruli served as a robust compensatory method for patients with insufficient IF findings.

In the present study, the exclusive focus on PLA2R, without considering other novel antigens (e.g., THSD7A, NELL1, EXT1/2), restricts the capacity for a comprehensive pathophysiological interpretation, particularly in seronegative cases. Secondly, the inability to evaluate IgG subtypes (IgG1-IgG4) prevented a detailed assessment of subtype-specific relationships between antibody response and tissue damage.

Given the retrospective single-center design, the generalizability of the findings is limited, and external validation in larger prospective multicenter cohorts is needed.

## Conclusion

Our single-center retrospective data suggest a potential discordance between circulating anti-PLA2R levels and glomerular PLA2R expression. These findings imply that a negative serum anti-PLA2R result may not entirely rule out tissue PLA2R positivity in every instance. Consequently, serum and tissue PLA2R might be better considered as complementary rather than mutually exclusive biomarkers, particularly in scenarios where clinical suspicion is high despite negative serology. In such cases, kidney biopsy followed by immunohistochemical evaluation may continue to serve as a supportive diagnostic tool.

## Data Availability

The datasets used in this study may be obtained by the corresponding author upon reasonable request and with the permission of the Pathology and Nephrology Clinics of Antalya Training and Research Hospital.
